# Supplementary material for: The gag-like gene RTL8 antagonizes PEG10-mediated virus like particles
Source: PLoS One. 2024 Dec 30;19(12):e0310946. doi: 10.1371/journal.pone.0310946 (PMC11684626; doi:10.1371/journal.pone.0310946)
Supplement: S1 File — (PDF) [file pone.0310946.s001.pdf]

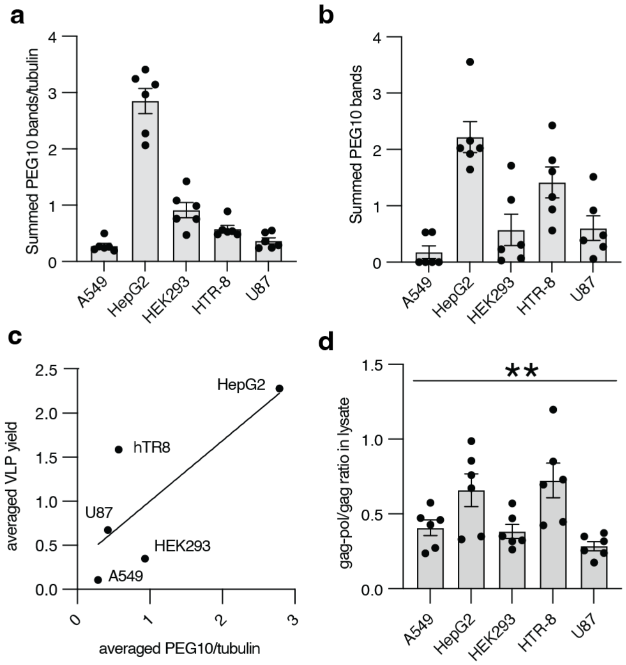

S1 Fig. Further quantitation of PEG10 in human cell lines. a-b) Quantification of total PEG10 abundance in lysate (a) and VLP fraction (b) for cells in Figure 1. Total PEG10 was calculated as the sum of gag-pol and gag signal normalized to tubulin. **c)** A simple linear regression of PEG10 VLP signal (y axis) and total PEG10 abundance in cell lysate (x axis). **d)** Quantification of PEG10 frameshift ratio in human cell lines. Frameshift ratio is calculated as gag-pol signal over gag signal. Data are analyzed by ordinary one-way ANOVA. p-values are corrected for multiple comparisons by Dunnett's test.

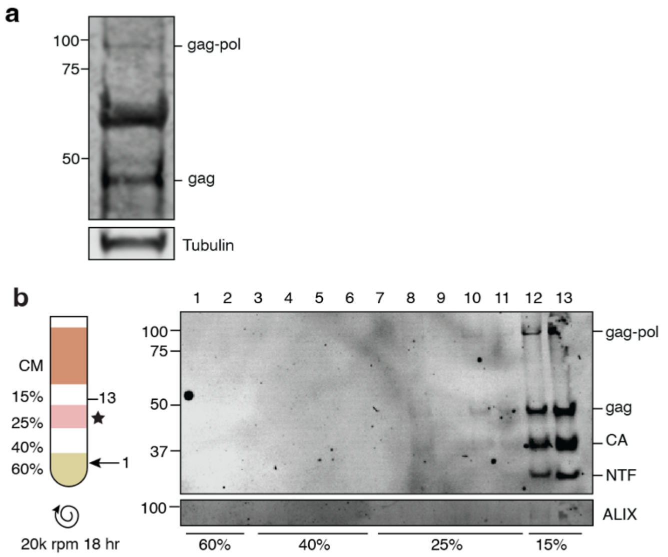

S2 Fig. Detection of PEG10-derived VLPs from induced neurons and transfected cells. a) Neurons were differentiated and cultured as in Fernandopulle et al. 2018. ~20mL cultured media was ultracentrifuged to generate the VLP fraction. Tubulin is observed in the VLP fraction, which may arise from Matrigel used during cell culture. **b)** Iodixanol fractionation of media supernatant from HEK293 cells overexpressing HA-PEG10. Left: schematic of iodixanol gradient preparation and harvest. Right: western blot of 13 consecutive fractions from iodixanol gradient showing PEG10 and ALIX.

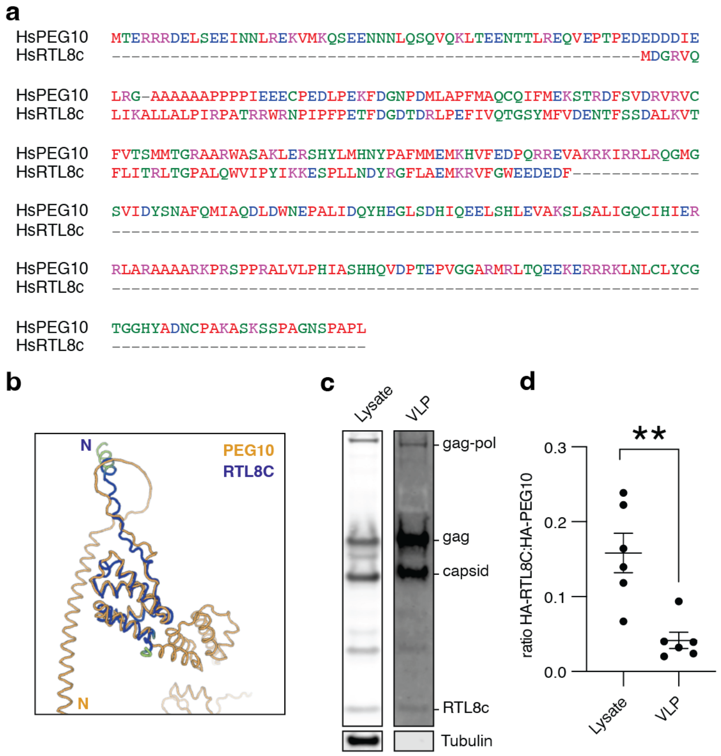

S3 Fig. PEG10 VLPs released into media contain a low level of RTL8 incorporation. a) Amino acid alignment of *Homo sapiens* PEG10 gag with RTL8C. **b)** DALI comparison of RTL8C and PEG10 gag show a high level of predicted structural homology. RTL8C and PEG10 gag were compared pairwise and structures overlaid using DALI, with RTL8C colored to show structural homology in blues, and PEG10 in orange. N-termini of proteins are annotated. **c)** Representative western blots of lysate (left) and VLP (right) samples prepared from cells expressing HA-tagged PEG10 and HA-tagged RTL8c. HA antibody was used to detect proteins, which were identified as either PEG10 or RTL8 based on molecular weight. **d)** Quantification of HA-RTL8c:HA-PEG10 ratio in lysate and VLP upon co-expression. RTL8 is less abundant in VLPs compared to lysate and is present in VLPs at less than 10% of PEG10 levels on average. Data were analyzed by Student's t-test. **p<0.01. Shown are data from 4 independent experiments.

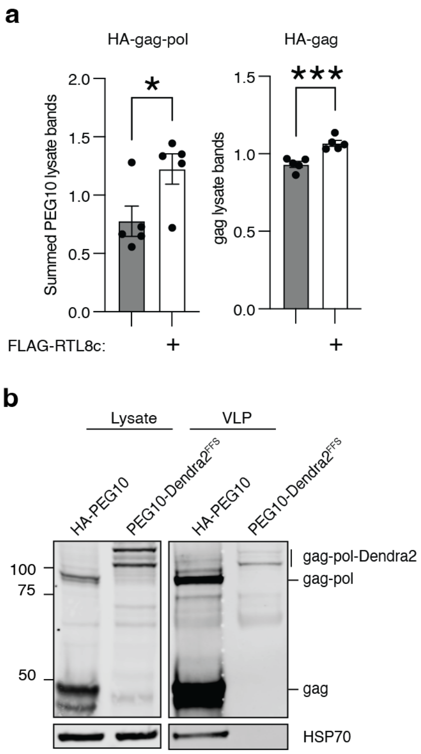

S4 Fig. Intracellular PEG10 levels accumulate upon RTL8c co-expression. a) Further quantification of PEG10 abundance in cell lysate with and without RTL8c co-expression. Left: cells transfected with PEG10 gag-pol; both gag and gag-pol bands were used to quantify intracellular PEG10 levels. Right: cells transfected with PEG10 gag. n = 5. **b)** Example western blot demonstrating that PEG10-Dendra2 is capable of forming VLPs that can be recovered from conditioned medium. Cells were transfected with either HA-PEG10 or a construct of PEG10-Dendra2 that only generates gag-pol protein (forced frameshift, 'FFS'), and VLPs were isolated as previously. Hsp70 band in HA-PEG10 VLP prep reflects contamination.

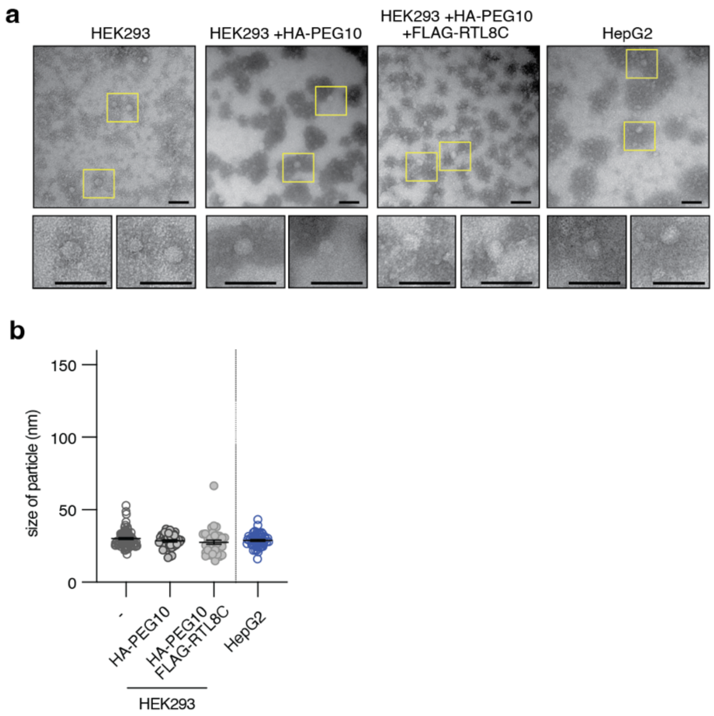

S5 Fig. Visualization and quantitation of particles isolated from 15% iodixanol fractionation of conditioned medium. a) Sample #13 from an iodixanol fractionation performed in Figure 2 was isolated for negative stain microscopy, as in Figure 3. Shown are representative images of at least 20 taken per sample, with particles imaged at higher magnification in yellow boxes. Scale bar = 100 nm. **b)** Length measurement of particles from each sample from (a) as in Figure 3. 75 particles were measured for HEK cells; 43 particles for HA-PEG10 transfected cells; 38 particles for HA-PEG10 and FLAG-RTL8C co-transfected cells; 53 particles for HepG2 cells.

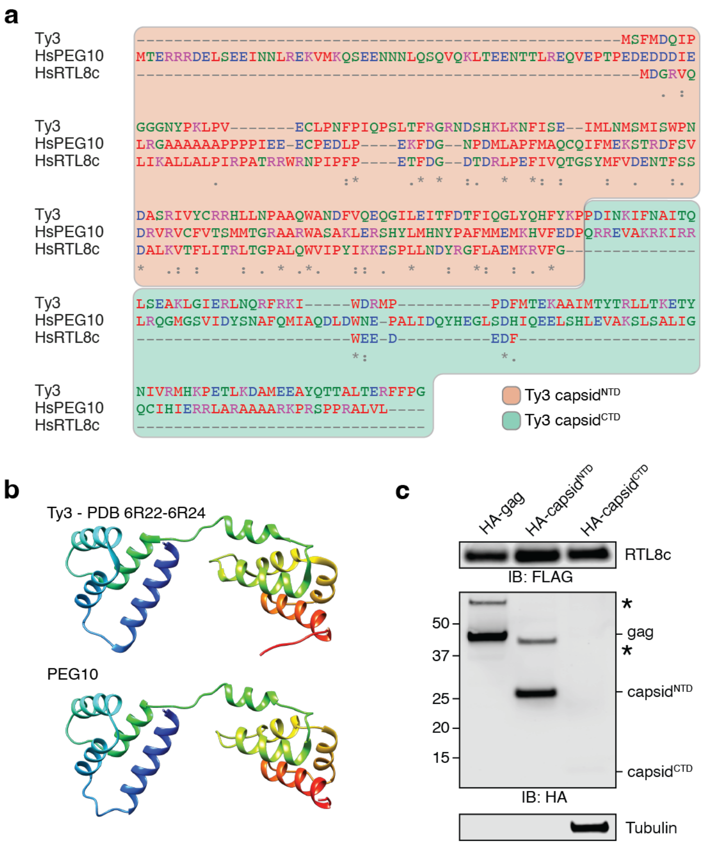

S6 Fig. PEG10 closely resembles Ty3 and self-associates in a manner resembling the ancestral retrotransposon. a) Sequence alignment of the Ty3 and PEG10 capsid domains, and full length RTL8c. The demarcation between Ty3 capsid^{NTD} and capsid^{CTD} is indicated with shaded color background. Amino acids are colored by property. **b)** (top) Structure of the Ty3 capsid. (bottom) Predicted structure of PEG10 gag. **c)** Co-IP of FLAG-RTL8c with HA-PEG10 gag, capsid^{NTD}, and capsid^{CTD}, as in Figure 3e. Five times the total input protein was loaded in the capsid^{CTD} immunoprecipitation to detect weak interactions with FLAG-RTL8c that may have been below the detection threshold in Figure 3e. Input concentrations were high enough that nonspecific interactions with tubulin were detected, but capsid^{CTD} remained below the limit of detection.

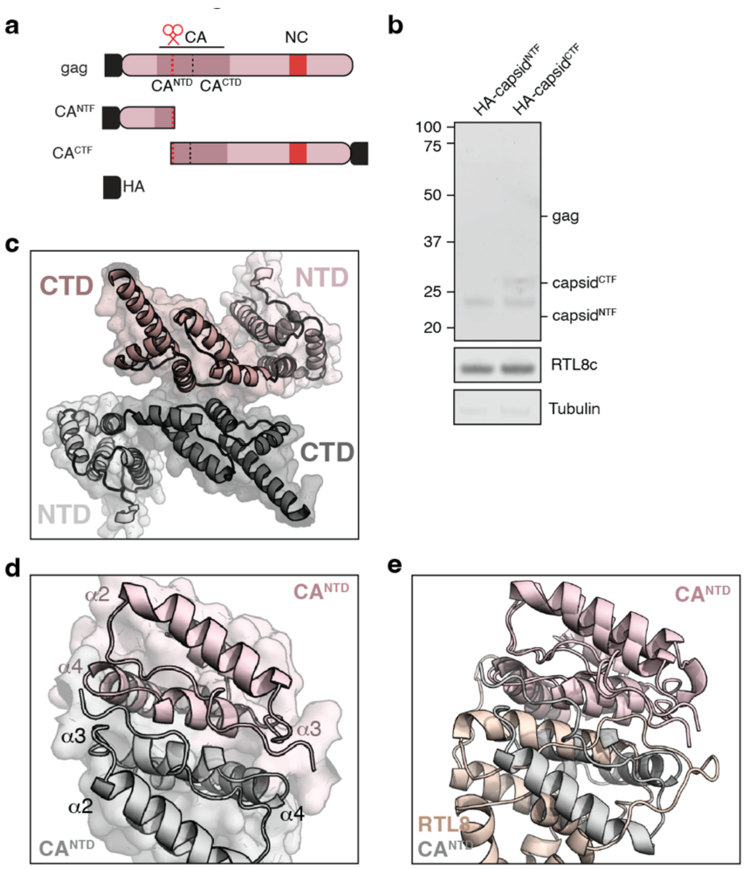

S7 Fig. PEG10 cleavage and modeling of binding. a) Diagram of additional PEG10 constructs including HA-N-terminal fragment and HA-C-terminal fragment products to approximate the endogenous cleavage event within the capsid. **b)** Co-immunoprecipitation of FLAG-RTL8c with capsid cleavage products HA-N-terminal fragment and HA-C-terminal fragment. **c)** Modeling of PEG10 capsid regions favors CTD:CTD interactions. One monomer of PEG10 is shown in pink, another in grey. NTD and CTD lobes are highlighted with different shades of color. **d)** Modeling two PEG10 NTD lobes together shows similar dimer structure to RTL8:PEG10. Alpha helices are numbered for each PEG10 monomer in pink and grey. **e)** Overlay of the PEG10 NTD:NTD structure with the RTL8:PEG10 structure.

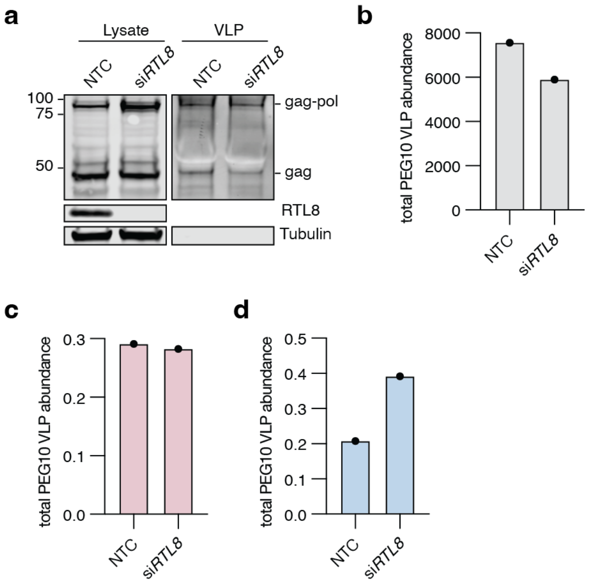

S8 Fig. Silencing of *RTL8* in hTR-8 cells does not improve PEG10 VLP yield. a) hTR-8 cells were transfected with siRNA against *RTL8* and conditioned medium was harvested 48 hr later for VLP western blot. n = 1. **b)** Quantitation of VLP abundance from Figure S7a. PEG10 VLP signal was measured as the sum of gag-pol and gag band intensities normalized as described in methods. **c-d)** Quantification of PEG10 gag (c), or gag-pol (d) abundance in cell lysate.
