# Supplementary material for: The gag-like gene RTL8 antagonizes PEG10-mediated virus like particles
Source: PLoS One. 2024 Dec 30;19(12):e0310946. doi: 10.1371/journal.pone.0310946 (PMC11684626; doi:10.1371/journal.pone.0310946)

Figure 1

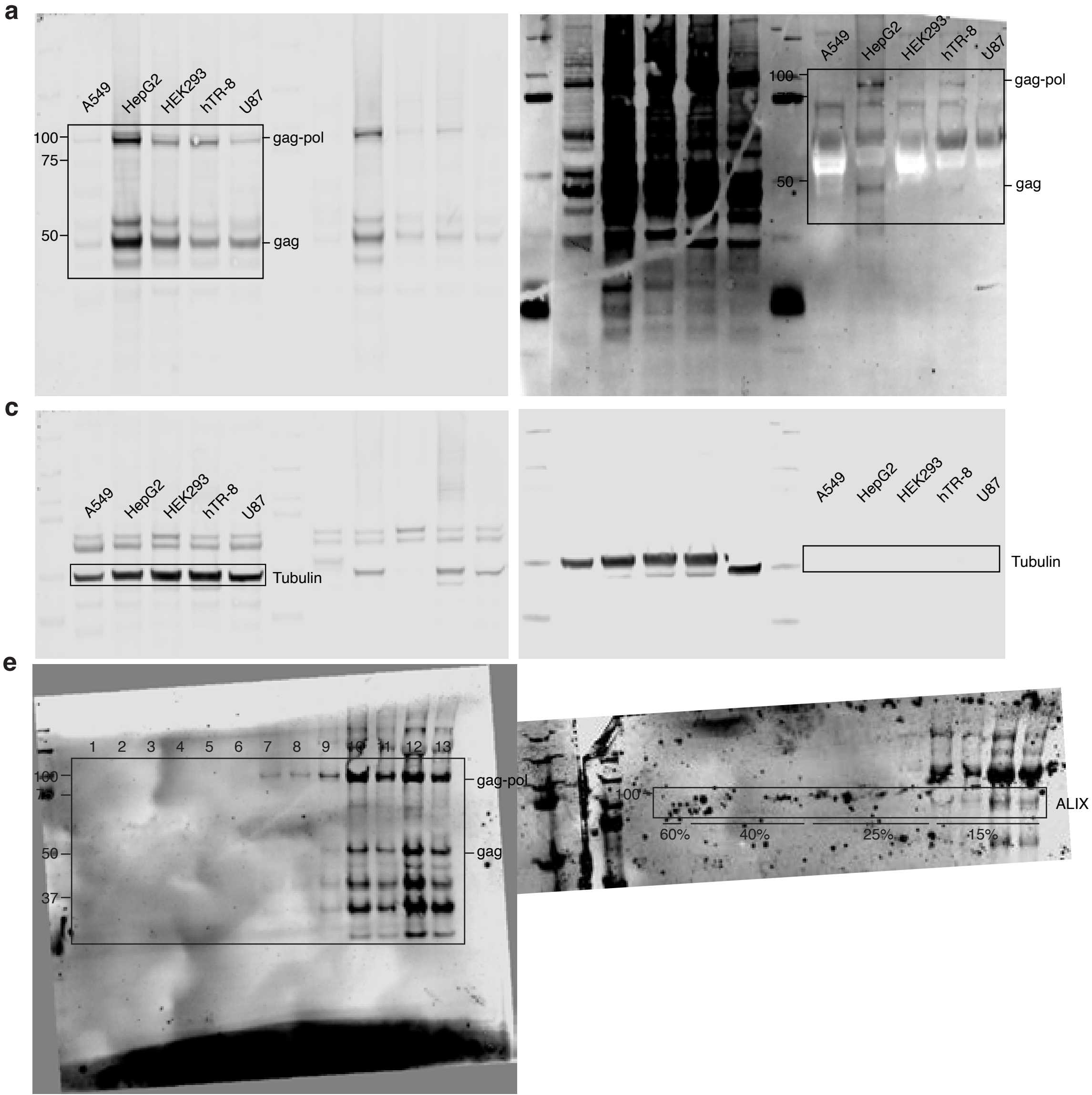
a
A549
HepG2
HEK293
hTR-8
U87
100
75
50
gag-pol
gag
c
Tubulin
e
1 2 3 4 5 6 7 8 9 10 11 12 13
37
ALIX
60%
40%
25%
15%

Figure 2

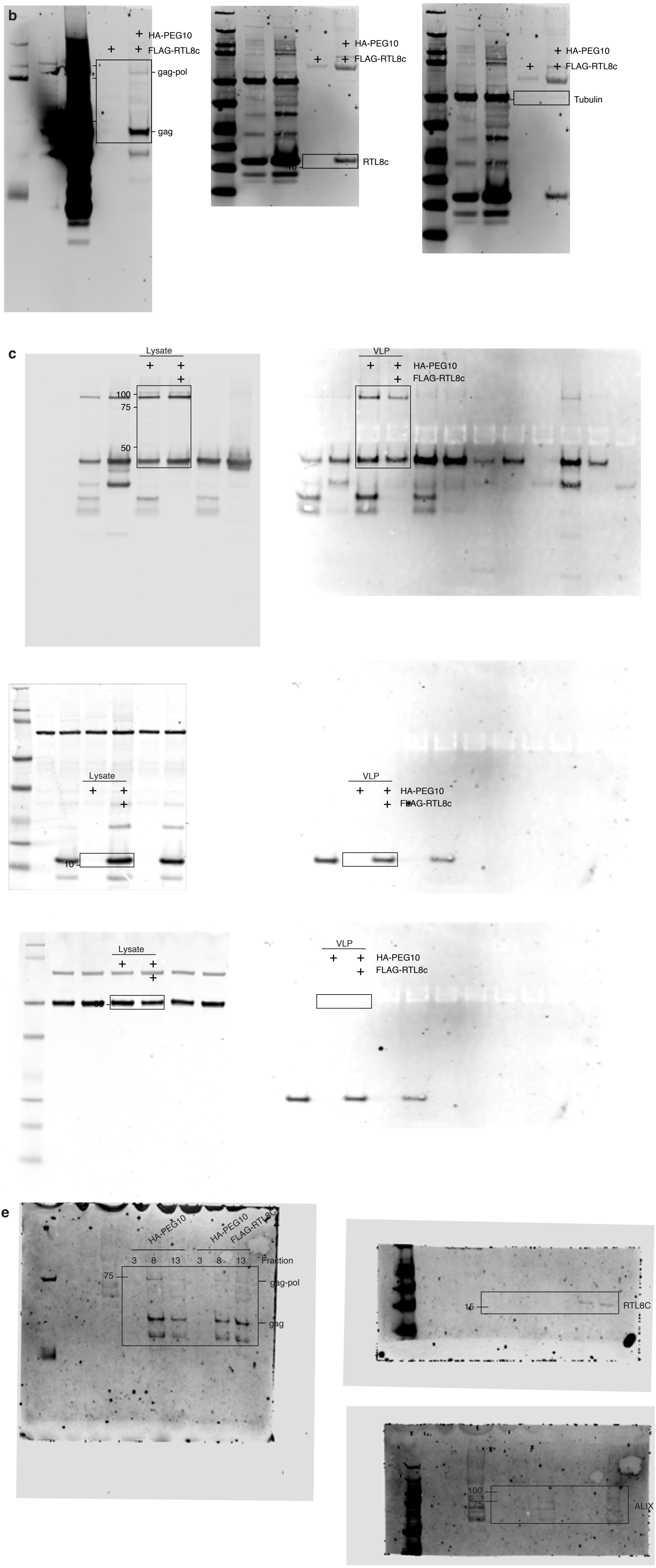
b
HA-PEG10
FLAG-RTL8c
gag-pol
gag
RTL8c
10
Tubulin
50
c
Lysate
VLP
100
75
50
10
e
HA-PEG10
FLAG-RTL8C
3
8
13
Fraction
75
gag-pol
gag
15
RTL8C
100
75
ALIX

Figure 5

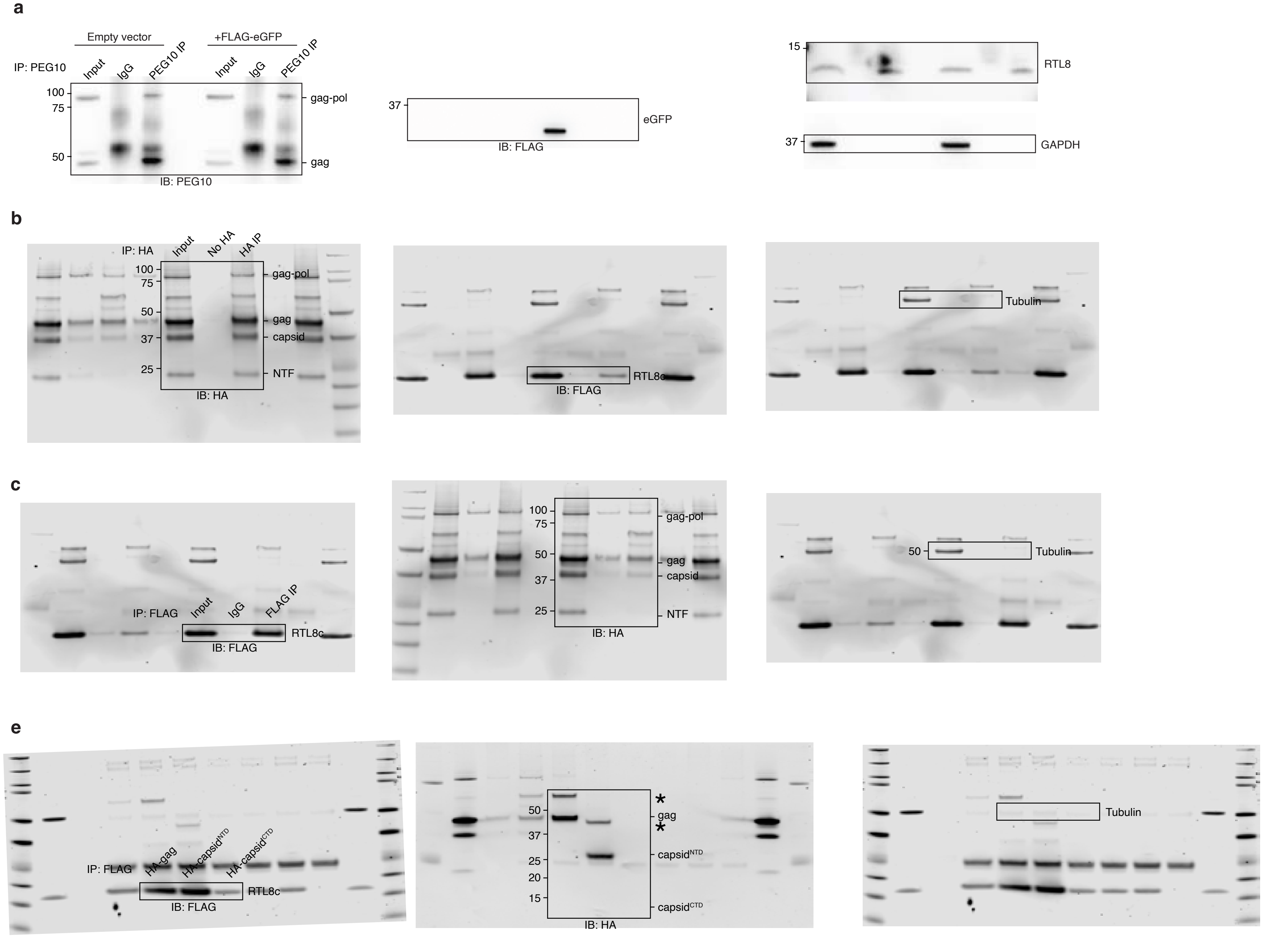

a
Empty vector
+FLAG-eGFP
IP: PEG10
Input
IgG
PEG10 IP
Input
IgG
PEG10 IP
100
75
50
gag-pol
gag
IB: PEG10
37
eGFP
IB: FLAG
15
RTL8
37
GAPDH
b
IP: HA
Input
No HA
HA IP
100
75
50
37
25
gag-pol
gag
capsid
NTF
IB: HA
RTL8c
IB: FLAG
Tubulin
c
IP: FLAG
Input
IgG
FLAG IP
RTL8c
IB: FLAG
100
75
50
37
25
gag-pol
gag
capsid
NTF
IB: HA
50
Tubulin
e
IP: FLAG
HA-gag
HA-capsid^NTD
HA-capsid^CTD
RTL8c
IB: FLAG
*
50
gag
*
37
25
capsid^NTD
20
15
capsid^CTD
IB: HA
Tubulin

Figure 6

c

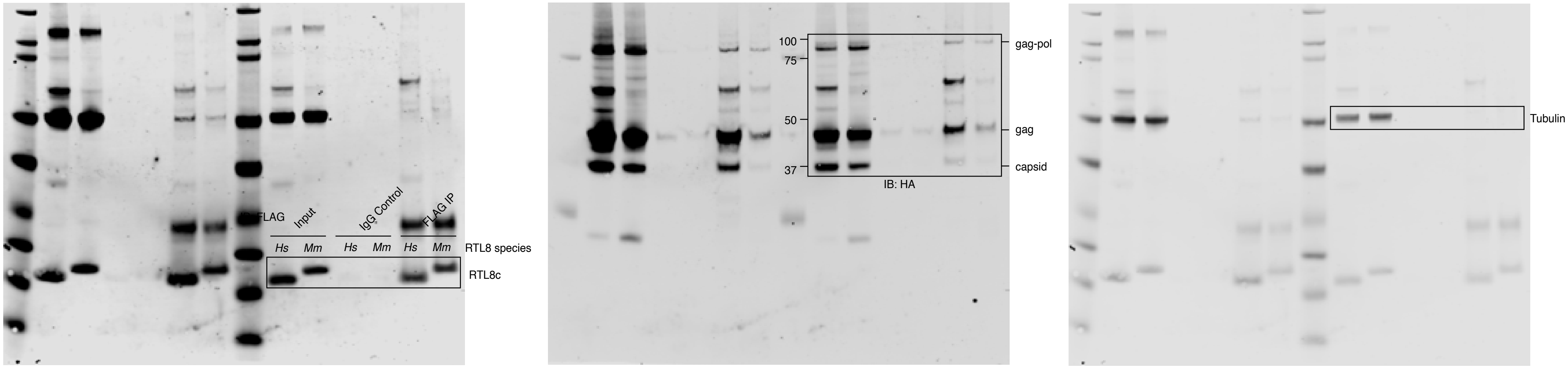

FLAG
Input
IgG Control
FLAG IP
Hs
Mm
Hs
Mm
Hs
Mm
RTL8 species
RTL8c
100
75
50
37
gag-pol
gag
capsid
IB: HA
Tubulin

e

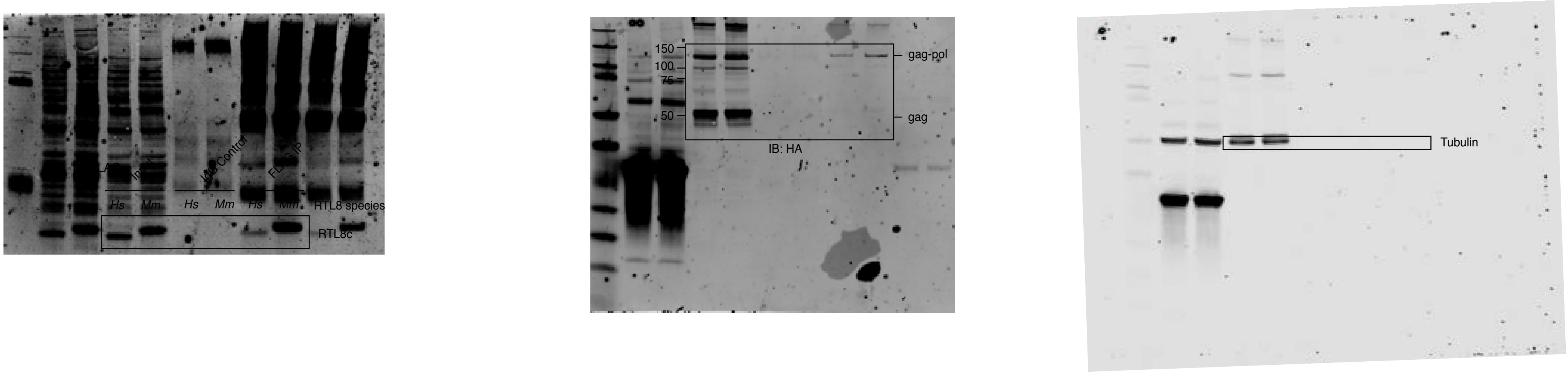

Input
IgG Control
FLAG IP
Hs
Mm
Hs
Mm
Hs
Mm
RTL8 species
RTL8c
150
100
75
50
gag-pol
gag
IB: HA
Tubulin

Figure 7

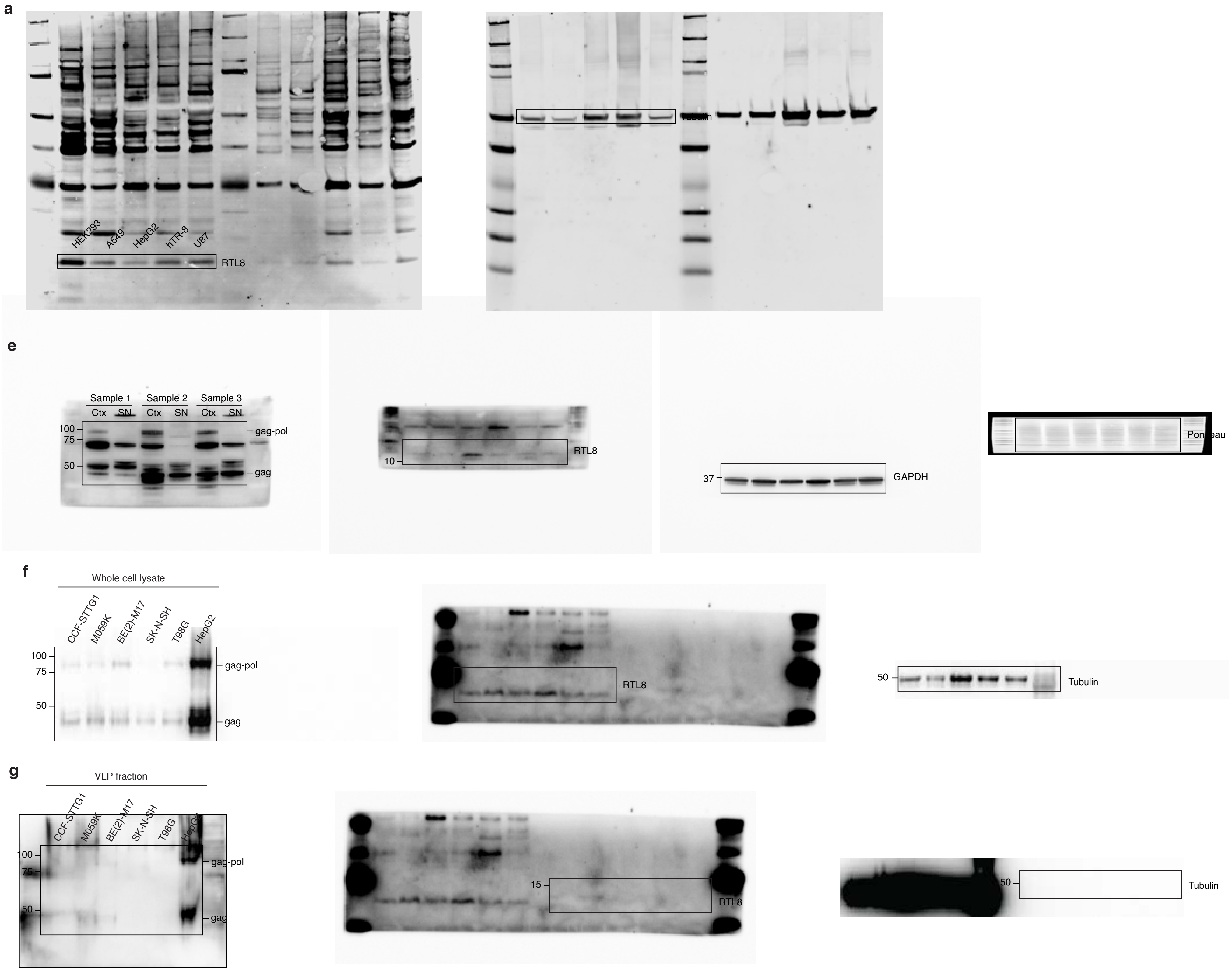
a
HEK293
A549
HepG2
hTR-8
U87
RTL8
Tubulin
e
Sample 1
Sample 2
Sample 3
Ctx
SN
Ctx
SN
Ctx
SN
100
75
50
gag-pol
gag
10
RTL8
37
GAPDH
Ponceau
f
Whole cell lysate
CCF-STTG1
M059K
BE(2)-M17
SK-N-SH
T98G
HepG2
100
75
50
gag-pol
gag
RTL8
50
Tubulin
g
VLP fraction
CCF-STTG1
M059K
BE(2)-M17
SK-N-SH
T98G
HepG2
100
75
50
gag-pol
gag
15
RTL8
50
Tubulin

Figure 8

a

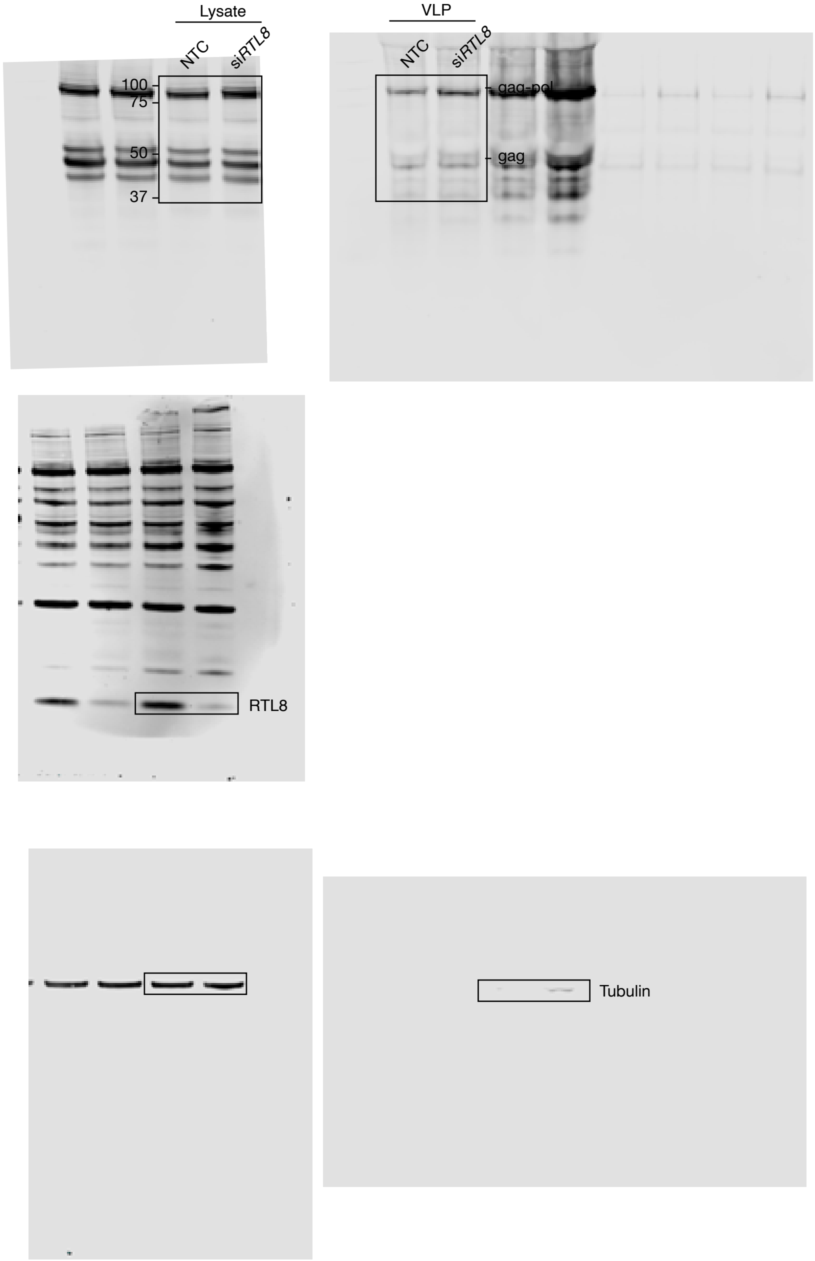

Lysate
NTC
siRTL8
VLP
NTC
siRTL8
100
75
50
37
gag-pol
gag
RTL8
Tubulin

Supplemental Figure 2

a

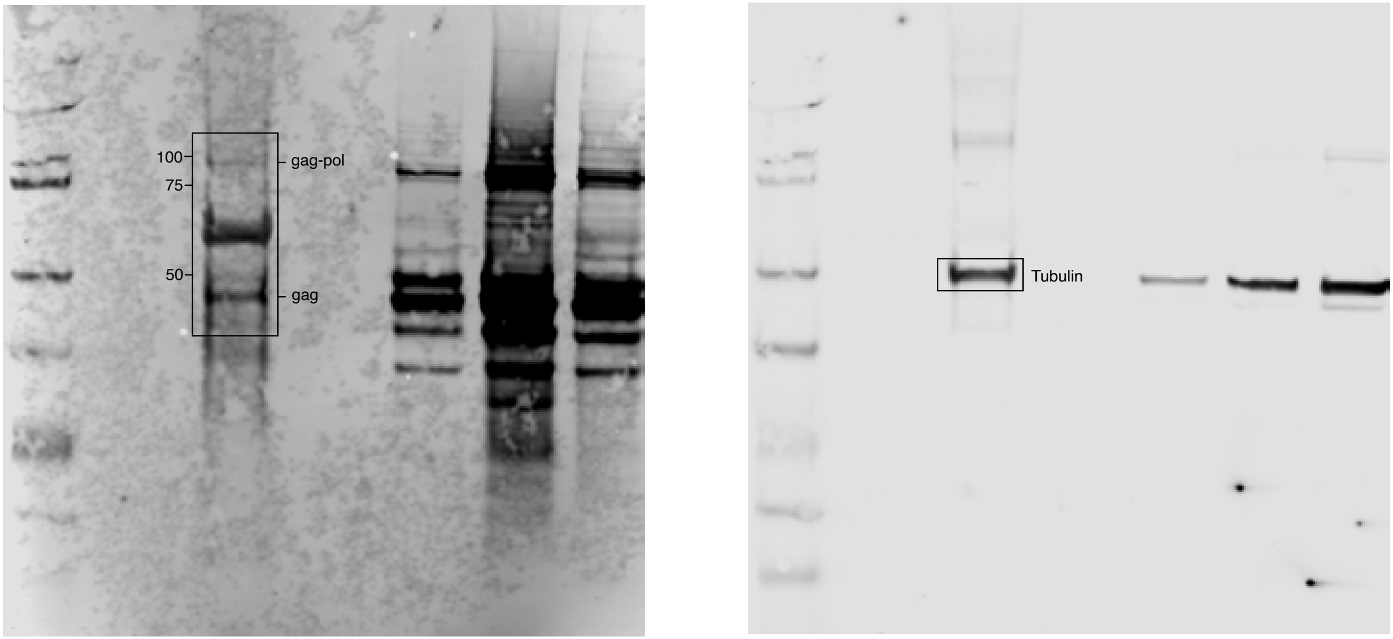

b

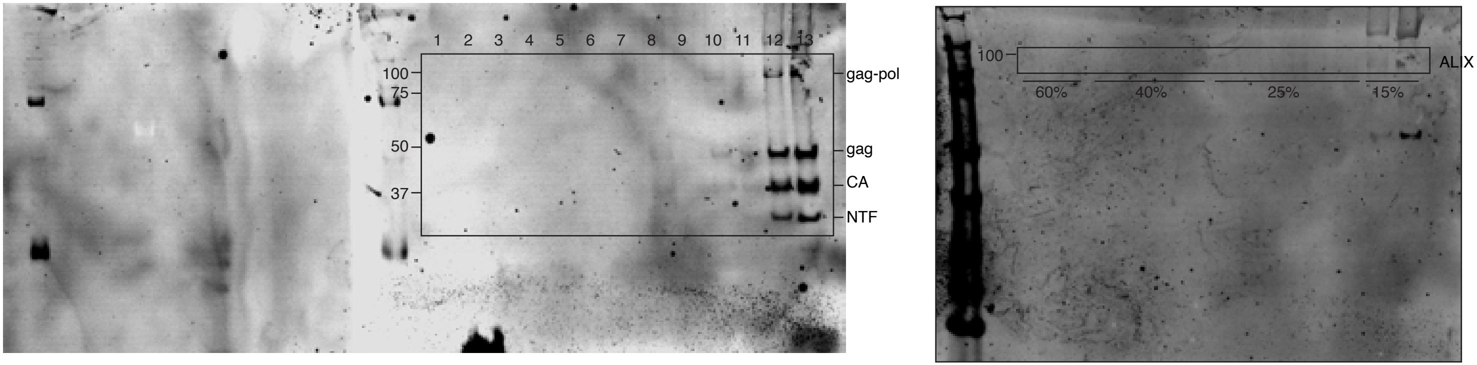

Supplemental Figure 3

c

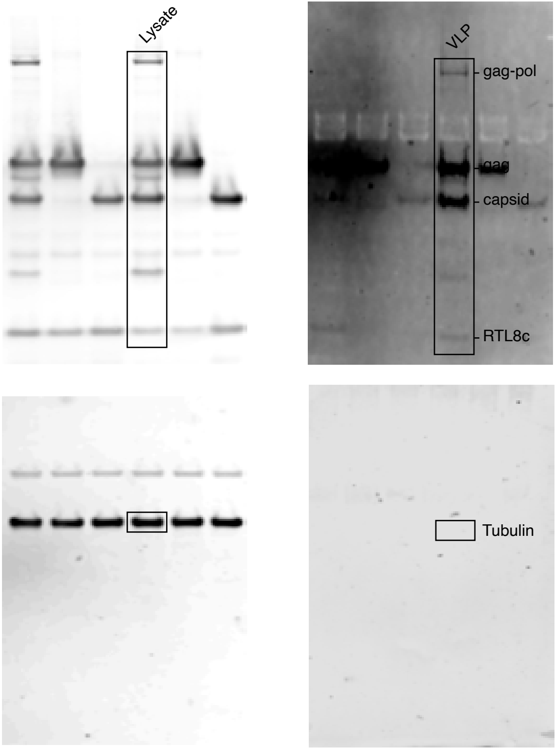

Supplemental Figure 4

b

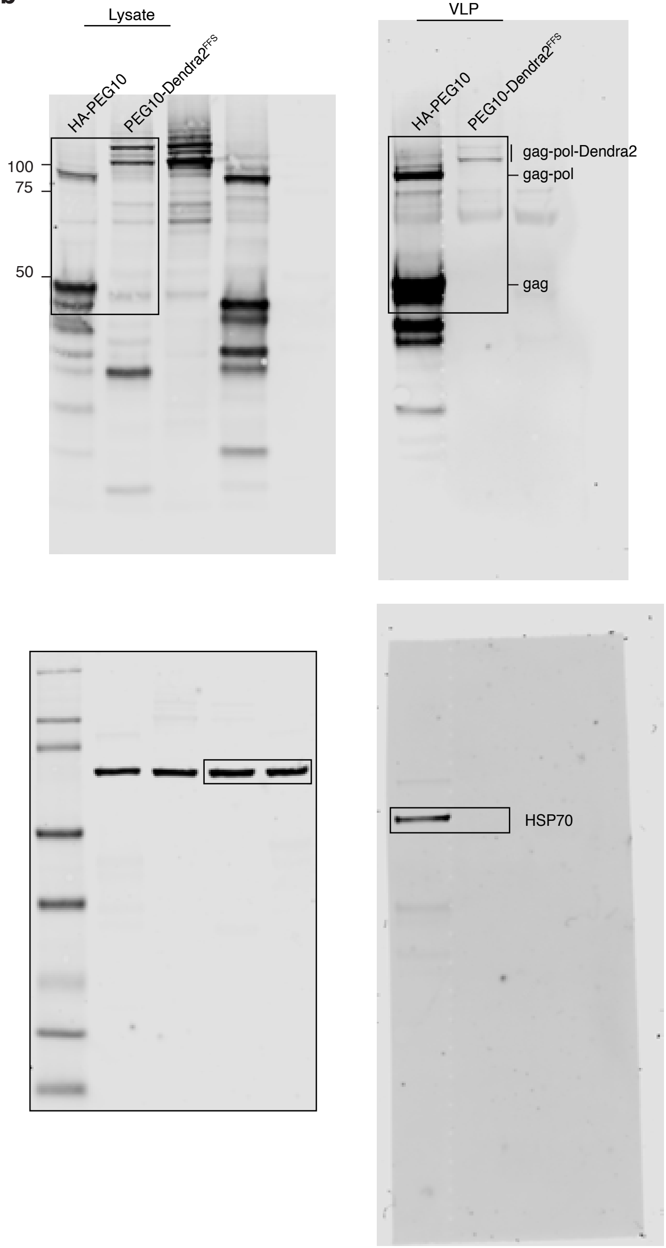

Supplemental Figure 6

c

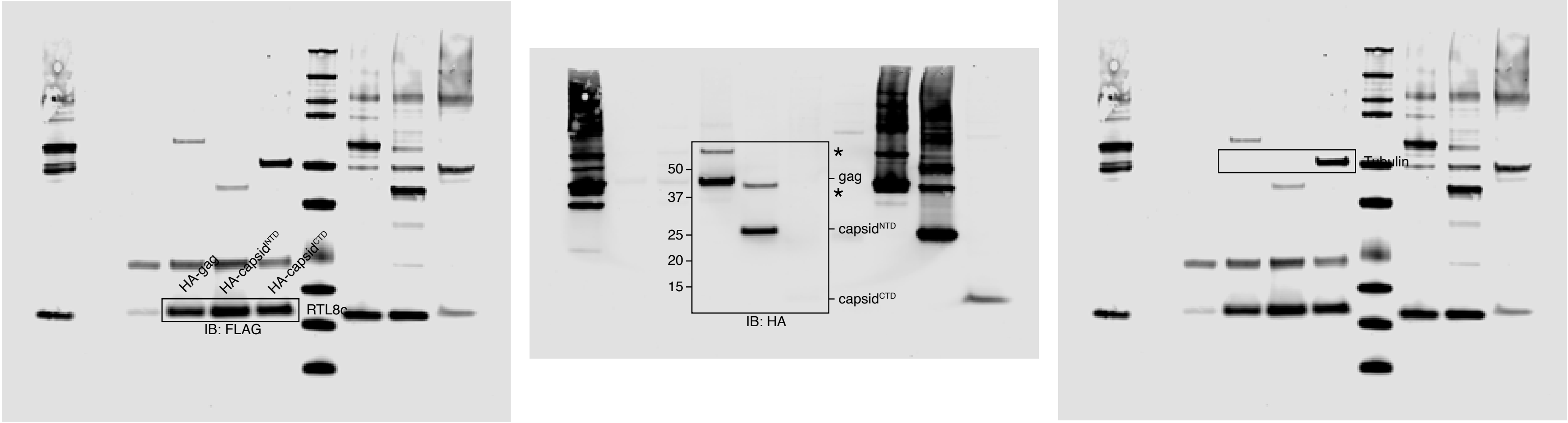

Supplemental Figure 7

b

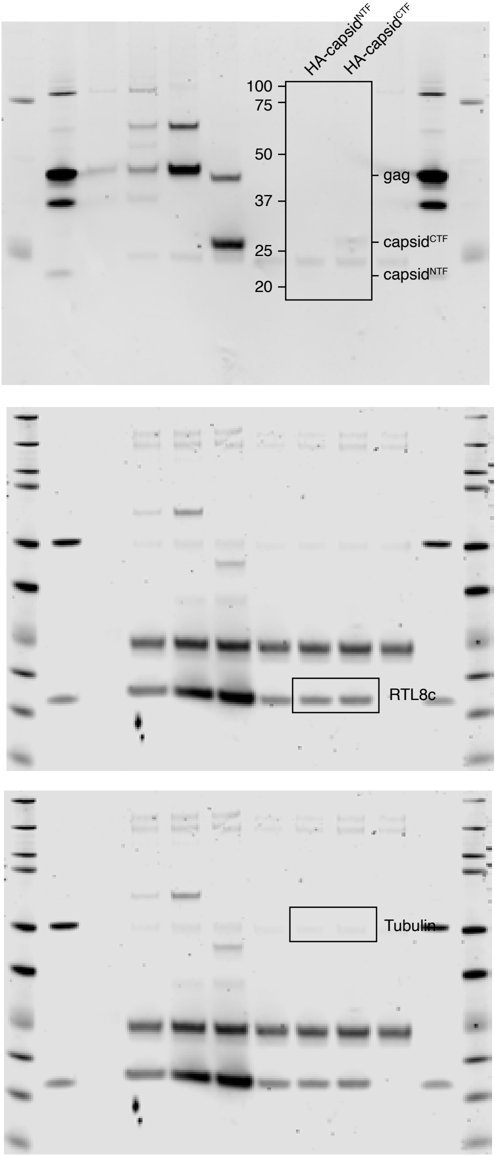

Supplemental Figure 8

a

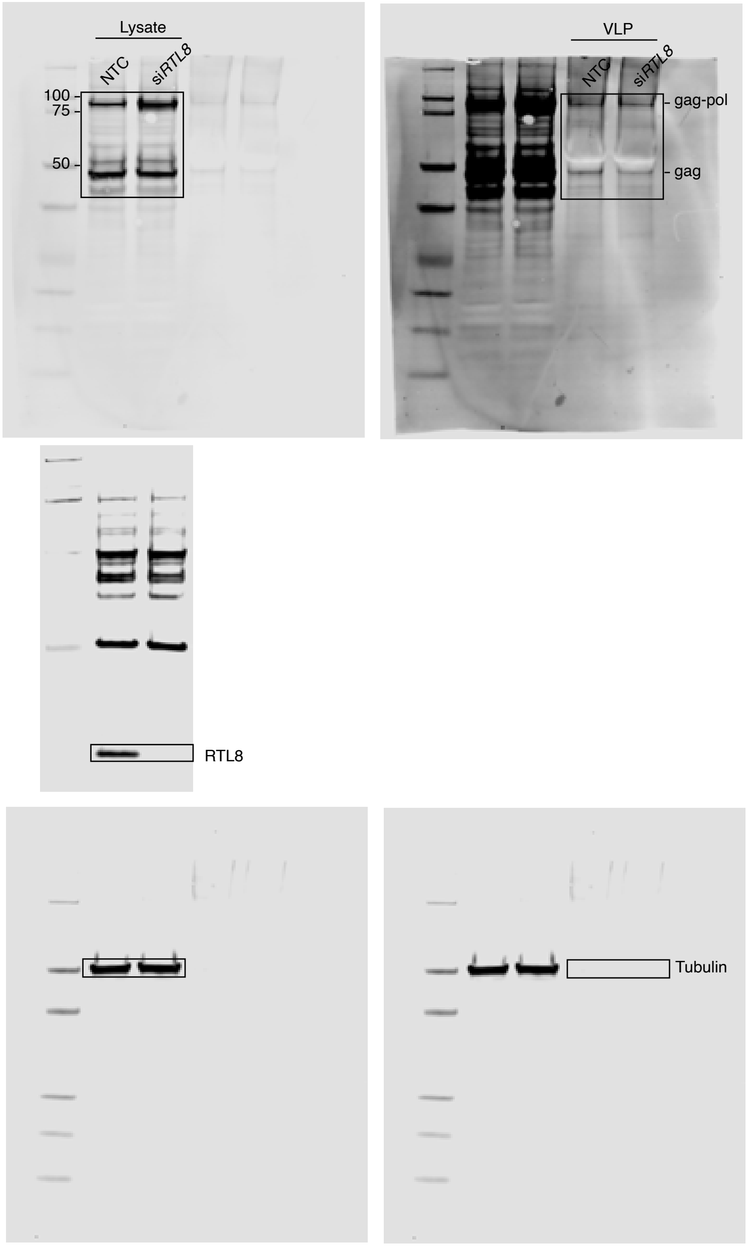

Supplement: S2 File — (PDF) [file pone.0310946.s002.pdf]
